# Can disc diffusion susceptibility tests assess the antimicrobial activity of engineered nanoparticles?

**DOI:** 10.1007/s11051-018-4152-3

**Published:** 2018-03-02

**Authors:** Angeliki Kourmouli, Marco Valenti, Erwin van Rijn, Hubertus J. E. Beaumont, Olga-Ioanna Kalantzi, Andreas Schmidt-Ott, George Biskos

**Affiliations:** 1 Faculty of Applied Sciences, Delft University of Technology, 2628-BL Delft, The Netherlands; 2 Department of Environment, University of the Aegean, 81100 Mytilene, Greece; 3 Present Address: The Birmingham Institute of Forest Research (BIFoR), School of Geography Earth and Environmental Sciences, University of Birmingham, Birmingham, B15 2TT UK; 4 Kavli Institute of Nanoscience, Department of Bionanoscience, Delft University of Technology, 2628-CJ Delft, The Netherlands; 5 Faculty of Civil Engineering and Geosciences, Delft University of Technology, 2628-CN Delft, The Netherlands; 6 Energy, Environment and Water Research Center, The Cyprus Institute, 2121 Nicosia, Cyprus

**Keywords:** Engineered nanoparticles, Disc diffusion method, Silver nanoparticles, Gold nanoparticles, Antimicrobial activity, Aerosol-based nanoparticle synthesis

## Abstract

The use of disc diffusion susceptibility tests to determine the antibacterial activity of engineered nanoparticles (ENPs) is questionable because their low diffusivity practically prevents them from penetrating through the culture media. In this study, we investigate the ability of such a test, namely the Kirby-Bauer disc diffusion test, to determine the antimicrobial activity of Au and Ag ENPs having diameters from 10 to 40 nm on *Escherichia coli* cultures. As anticipated, the tests did not show any antibacterial effects of Au nanoparticles (NPs) as a result of their negligible diffusivity through the culture media. Ag NPs on the other hand exhibited a strong antimicrobial activity that was independent of their size. Considering that Ag, in contrast to Au, dissolves upon oxidation and dilution in aqueous solutions, the apparent antibacterial behavior of Ag NPs is attributed to the ions they release. The Kirby-Bauer method, and other similar tests, can therefore be employed to probe the antimicrobial activity of ENPs related to their ability to release ions rather than to their unique size-dependent properties.

**Graphical abstract:**
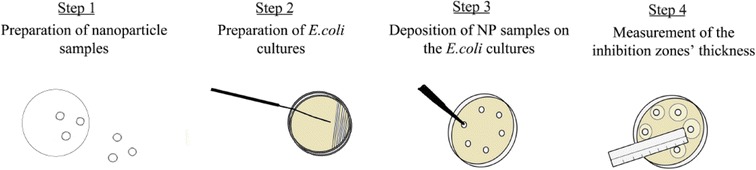
ᅟ

ᅟ

## Introduction

Engineered nanoparticles (ENPs) have numerous applications in medicine including treatments for fungal and HIV infections, as well as various types of cancer (Zhang et al. 2008). Compared to their large-particle counterparts, ENPs also exhibit enhanced antimicrobial activity and are therefore employed in many hygiene products (Choi et al. 2008; Sotiriou and Pratsinis 2010). A number of methods, including the Kirby-Bauer disc diffusion susceptibility technique, have been used to determine the antibacterial activity of ENPs (Oberdörster et al. 2005). The challenge of assessing the toxicity of ENPs, however, lies in the difficulty to discriminate between size-related effects caused by direct particle–cell interactions, from those caused by the potential compounds they release upon dissolution to the culture media.

An increasing number of studies have employed the Kirby-Bauer method for assessing the antibacterial activity of ENPs. Guzman et al. (2012) as well as Devi and Bhimba (2012) used this technique to show that Ag nanoparticles (NPs) smaller than 30 nm produced by standard liquid-based methods can have strong antibacterial effects. Geethalakshmi and Sarada (2012) used the same method to show that Ag and Au NPs larger than 30 nm produced by extracts from *Trianthema decandra* roots also have enhanced antimicrobial activity. More recently, Bhuyan et al. (2017) used the Kirby-Bauer technique for assessing the antimicrobial activity of Ag and Au NPs produced by extracts from *Paederia foetida Linn.* Their results showed that Au NPs did not exhibit any antimicrobial activity against all pathogens, contrary to their Ag counterparts. It should be noted here that these studies do not distinguish between the toxic behavior caused by direct nanoparticle–cell interactions and those induced by potential dissolution of toxic species from the surface of the NPs. This point is particularly important for the interpretation of the results since, as will be demonstrated below, only species released from the NPs can penetrate into the culture media and inhibit the growth of the cells.

The Kirby-Bauer method relies on the diffusion of the test substance (i.e., the ENPs in the aforementioned studies) from the filter discs to the bacterial cultures (cf. Methods section for more details). The diffusivity of NPs having diameters larger than 10 nm in culture media used in diffusion susceptibility tests is in the order of 10^−11^ m^2^/s. This is at least one order of magnitude lower compared to the respective diffusivity of common antibiotics for which such tests are commonly used. As a consequence, ENPs do not travel far from the deposition discs to physically interact with the bacterial cells, raising doubts whether the method can probe antibacterial activity related to their size. To the best of our knowledge, this has not been considered by other studies reported in the literature thus far.

The aim of this study is to test the hypothesis that the Kirby-Bauer method only detects antibacterial effects of ENP-derived dissolved compounds. To this end, we examined the antibacterial activity of pure ENPs composed of Au and Ag (i.e., two metals that behave differently in aqueous media but have a toxic behavior at the nanoscale; cf. Sadeghi et al. 2010; Peretyazhko et al. 2014; Ilaria et al. 2015; Shrivastava et al. 2016; Chandran et al. 2017) on *Escherichia coli* cultures. ENPs had diameters from 10 to 40 nm, and to make the results comparable among all tests, we kept their total surface concentration constant in all our samples.

## Methods

### Particle production

**Fig. 1:**
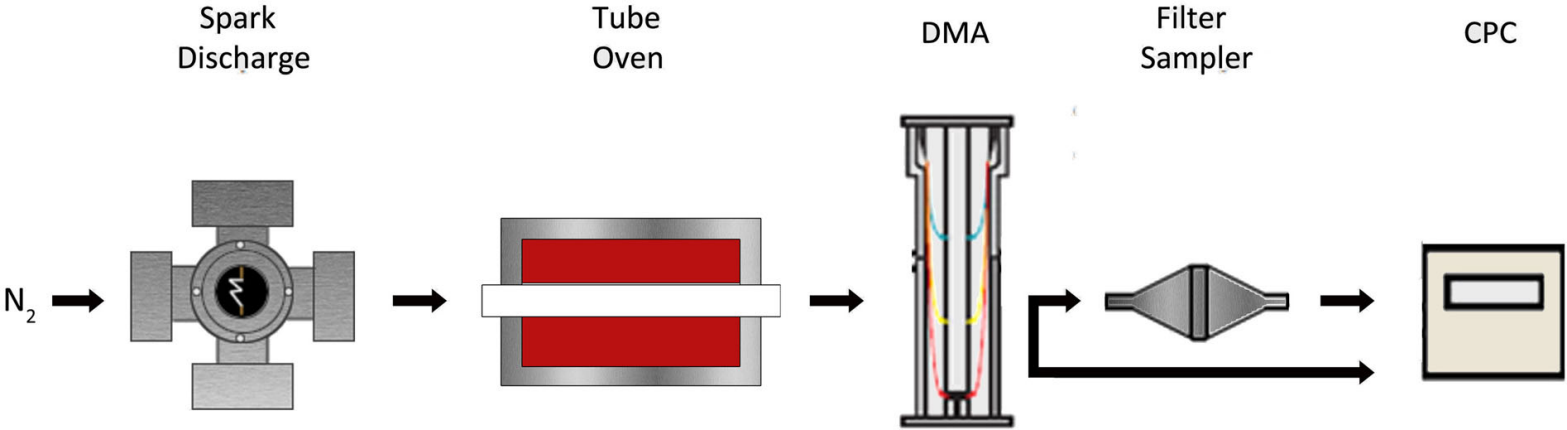
Schematic layout of the apparatus used for the production of ENPs. High-purity Au or Ag agglomerates were produced by spark ablation and sintered to spherical particles in a tube oven. Monodisperse fractions of the resulted spherical particles were selected by a DMA and deposited on glass fiber filters. The concentration of the monodisperse particles downstream the DMA and the filter sampler was continuously monitored by a CPC

Pure (ligand-free) Au and Ag NPs were synthesized by vapor nucleation in N_2_ gas (99.999% purity) using a spark-discharge particle generator (cf. Fig. 1). This method, described in detail by Tabrizi et al. (2009) and more recently by Pfeiffer et al. (2014), can be used to synthesize well-defined NPs with good control over their composition, including both single-component or mixed/alloy NPs of high purity (Feng et al. 2018). What is also important for employing this technique to produce samples for toxicity tests is that combined with a Differential Mobility Analyzer (DMA; i.e., a classifier that selects particles based on their electrical mobility; Knutson and Whitby 1975), it can produce uniformly-sized NPs having diameters within a very narrow range (i.e., nearly monodisperse NPs) as has been illustrated by a number of recent studies (Feng et al. 2015; Feng et al. 2016; Valenti et al. 2017).

**Fig. 2:**
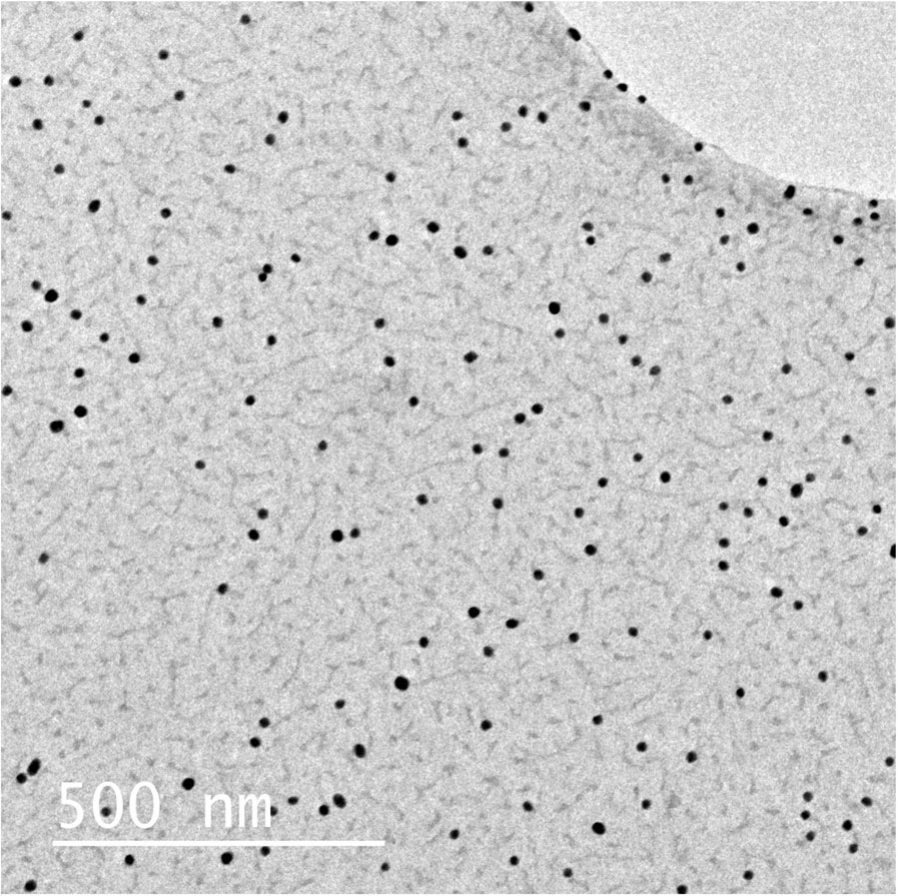
Transmission electron microscope image of the nearly-monodisperse Ag NPs produced by the experimental setup used in this study

In brief, two opposing cylindrical Ag or Au electrodes (MaTecK GmbH, Germany; 99.99% purity) are placed a few millimeters apart. Repeated electrical breakdowns form when a high potential difference is applied between the two electrodes, resulting in a nearly-continuous evaporation of material from their surface. A high-purity N_2_ gas flow passed between the two electrodes dilutes and cools the vapor cloud to form pure Ag or Au NPs of different sizes and morphologies (i.e., from spherical to highly agglomerated particles) depending on the flow rate and electrical energy used to form the sparks (Feng et al. 2015). A tube oven maintained at ~ 1000 °C was used downstream the spark-discharge generator to sinter the agglomerates to spherical particles. An example Transmission Electron Microscopy (TEM) image of 15-nm Ag NPs produced by the setup used in this study is provided in Fig. 2, verifying the capability of the system to produce nearly monodisperse spherical particles. Similar results were obtained for NPs having sizes within the entire range invested here, including for the Au NPs. In addition to that, X-ray photoelectron spectroscopy confirmed that the resulting NPs consisted purely of Au or Ag depending on the electrodes used in the spark-discharge generator (data not shown here). Compared to classical wet-chemistry methods for synthesizing ENPs, spark ablation in the gas phase delivers NPs of higher purity (Biskos et al. 2008), which is crucial for studying their interactions with living cells.

To select particles of a specific size, we used a custom-made DMA having an effective length of 114 mm and diameters of the outer and inner electrodes of 19.54 and 9.35 mm, respectively (cf. Barmpounis et al. 2016, and the supplementary information therein, for additional details). After size selection, the particles were collected on glass-fiber filter discs with a diameter of 47 mm. A Condensation Particle Counter (CPC; TSI Model 3025; Agarwal and Sem 1980) was used to measure the aerosol nanoparticle concentration in order to control the total surface area of the collected particles, which was kept constant for all the filters prepared (i.e., ~ 14.5 × 10^−5^ m^2^ for both Ag and Au ENPs).

### Toxicity experiments

**Fig. 3:**
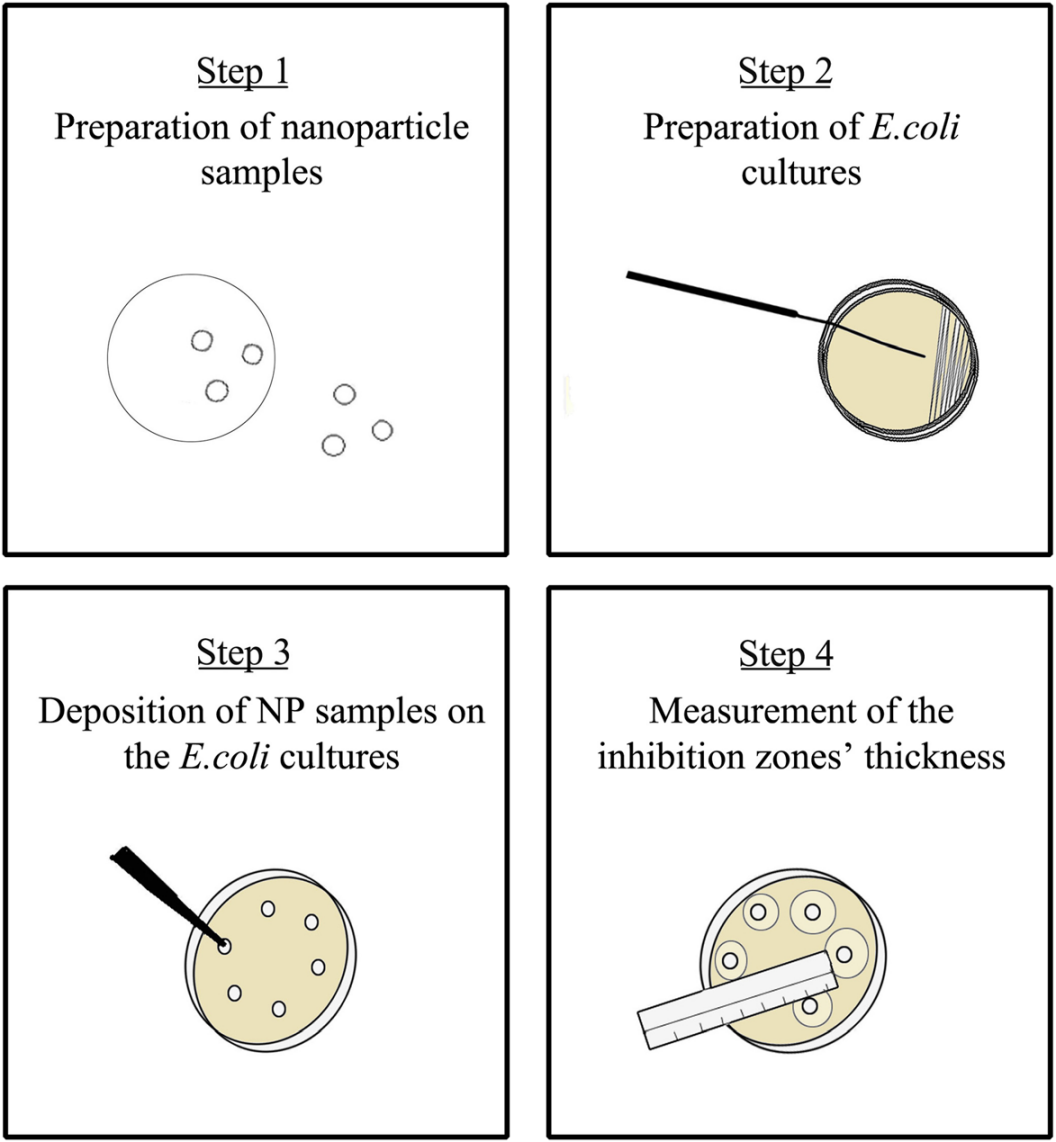
Schematic layout of the experimental procedure used for the toxicity experiments. Five-millimeter nanoparticle-laden filter discs were cut out of the original filter (step 1) while *E. coli* cultures were laid on the MH2 agar plates (step 2). The filter discs were then deposited on the *E. coli* cultures with the nanoparticle deposition side facing down (step 3). The size of the inhibition zones around the filter discs was measured after 48 h of incubation (step 4)

Cultures of *E. coli* were cultivated on Mueller-Hinton (MH2) agar plates as illustrated in Fig. 3. Bacteria samples from one or two over-night grown colonies were suspended in a test tube containing nutrient broth (i.e., lysogeny broth). The turbidity (expressed as optical density; OD) of the bacterial suspensions were measured with an optical spectrophotometer (λ = 600 nm) and adjusted to 0.25 or 0.50. A sterilized cotton swab was immersed in the resulting suspension, and a lawn of bacteria was applied on the agar plates.

Nanoparticle-laden filter discs with a diameter of 5 mm were cut out of the 47-mm filters and applied on top of the bacterial cultures. The side containing the particles was facing downwards to ensure direct interaction with the agar and the bacteria, and the samples were incubated at 37 °C for 48 h. The inhibition zones around the filters were measured every 12 h according to the Kirby-Bauer test protocol (Bauer et al. 1966).

## Results and discussion

**Fig. 4:**
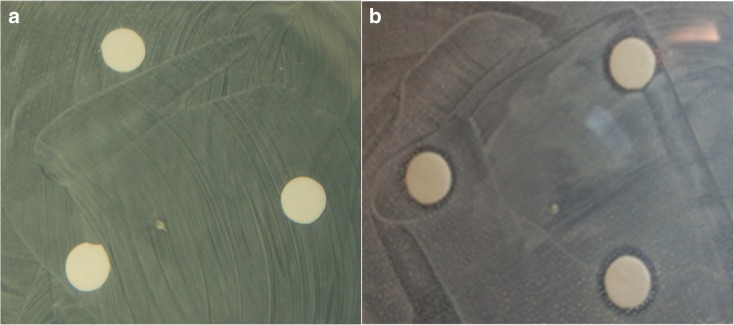
Images of grown *E. coli* cultures with ENP-laden filter discs containing 10-nm Au (**a**) and Ag (**b**) ENPs. In both cases, the surface concentration of the particle depositions was the same (i.e., ~ 14.5 × 10^−5^ m^2^)

Table 1 shows the size of the inhibition zones around the filters loaded with Ag and Au ENPs having diameters of 10, 20, and 40 nm. Figure 4 shows images of the *E. coli* cultures with the deposited ENP-laden filter discs after 48 h. The cultures exposed to Au NPs did not exhibit any inhibition zones around the filters, indicating that they do not have any antibacterial properties. The tests with the Ag NPs, on the other hand, exhibited inhibition zones of the same size for all particle diameters tested. It should be noted here again that the total surface of the particles on the filter samples was kept the same in order to ensure that the potential release rates of the ions (or of any other substance from the surface of the NPs) was the same throughout all tests (cf. Liu and Hurt 2010; Lee et al. 2012).

**Table 1:** Size of inhibition zones (mm) measured from the edge of the filter discs to the edge of the bacterial lawn, around the nanoparticle-laden filters

Particle	Ag Nanoparticles		Au Nanoparticles	
Diameter (nm)	(OD: 0.5)	(OD: 0.25)	(OD: 0.5)	(OD: 0.25)
10	2	1	0	0
20	2	1	0	0
40	2	1	0	0

Although the results summarized in Table 1 can be interpreted as a difference in the antibacterial effects between Ag and Au ENPs on *E. coli*, they should be treated with caution. The most plausible explanation of our observations is related to the release of ions from the ENPs. In contrast to Au, Ag releases ions upon oxidation and suspension in aqueous solutions (e.g., Wijnhoven et al. 2009; Bae et al. 2010; Le Ouay and Stellacci 2015). Considering that Ag^+^ ions have a strong antimicrobial activity (Zhao and Stevens 1998; Feng et al. 2000; Xiu et al. 2012) that can inhibit bacterial growth, the observed discrepancies between the measurements using Ag and Au NPs can be attributed to the difference of this intrinsic property for the two materials.

The hypothesis that the Ag^+^ ions are the antimicrobial agents in these tests is also consistent with the fact that the size of the inhibition zones around the Ag-nanoparticle-laden filters was the same for all particle sizes tested. This is because the total nanoparticle surface concentration was kept constant throughout all samples, ensuring that the ion release rate was the same in all the tests as discussed above. Additional supporting evidence that the Ag^+^ ions are responsible for inhibition of bacterial growth in these tests is the actual size of the inhibition zones around the Ag-nanoparticle filters. Considering that those were up to a couple of millimeters wide, this observation would require a toxic species having a diffusivity in the order of 10^−9^ m^2^/s in the solution of the culture. This value is of the same order as the diffusivity of silver ions in the culture medium, and definately much higher than that of ENPs larger than 10 nm that are used in our experiments.

In conclusion, our observations show that the Kirby-Bauer method (and in principle other similar disc diffusion susceptibility tests) can be used for assessing the antimicrobial activity of ENPs. It should be noted, however, that the probed antibacterial activity is related to the potential of the NPs to release toxic and highly diffusive ions (or other soluble species) into the culture growth medium, which in the case of our experiments was due to the dissolution of Ag, rather than the physical interaction with the nanometer-sized particles. This characteristic can explain the difference in the antibacterial behavior of Au and Ag ENPs tested in this study, and can be used in a systematic way for distinguishing between ion and nanosize toxicity of ENPs.
